# Thermal decomposition of propylene oxide with different activation energy and Reynolds number in a multicomponent tubular reactor containing a cooling jacket

**DOI:** 10.1038/s41598-022-06481-4

**Published:** 2022-03-09

**Authors:** Abid A. Memon, M. Asif Memon, Kaleemullah Bhatti, Ilyas khan, Nawa Alshammari, Amnah S. Al-Johani, Nawaf N. Hamadneh, Mulugeta Andualem

**Affiliations:** 1 Department of Mathematics and Social Sciences, Sukkur IBA University, Sukkur, 65200 Sindh Pakistan; 2 Department of Mathematics and Statistics, Faculty of Applied Sciences and Technology, Universiti Tun Hussein Onn, Malaysia Batu Pahat, 86400 Johar, Malaysia; 3 Department of Mathematics, College of Science Al-Zulfi, Majmaah University, Al-Majmaah, 11952 Saudi Arabia; 4 Department of Basic Sciences, College of Science and Theoretical Studies, Saudi Electronic University, Riyadh, 11673 Saudi Arabia; 5 Mathematics Department, Faculty of Science, University of Tabuk, Tabuk, Saudi Arabia; 6 Department of Mathematics, Bonga University, Bonga, Ethiopia

**Keywords:** Chemistry, Mathematics and computing, Engineering

## Abstract

In this article, we are focusing on heat and mass transfer through a Multicomponent tubular reactor containing a cooling jacket by thermal decomposition of propylene oxide in water. The chemical reaction is an irreversible, 1st order reaction and an exothermic reaction that yields propylene glycol with enthalpy = −84,666 J/mol. The constant rate of the reaction is followed by the Arrhenius equation in which the activation energy is taken on a trial basis in the range from 75,000 to 80,000 J/mol with a fixed frequency factor. For the fluid to flow, the Reynolds number is kept in the range from 100 to 1000. The three partial differential equations of mass, momentum, and energy are coupled to study heat and mass transfer in a tubular reactor by using the chemistry interface in COMSOL Multiphysics 5.4. The initial concentration of propylene oxide is tested in the range from 2 to 3% and the thermal conductivity of the mixture is tested in the range 0.599–0.799. It was found that the amount deactivated of the compound decreases with an increase in Reynolds number. Propylene oxide is decomposed at about 99.8% at *Re* = 100 at lower activation energy and gives the total maximum enthalpy change in the tubular reactor. Observing the relationship between Sherwood numbers to Nusselt numbers, it was deducted that the convective heat transfer is opposite to convective mass transfer for high Reynolds numbers.

## Introduction

Multicomponent tubular reactors as vessels are widely used to investigate the heat and mass transfer problems under the chemical reactions at different boundary conditions^1–4^. Also, millions of chemical species interacting with water are used to model and optimize the thermal distribution and mass distribution in tubular reactors in many industrial applications to make the required product quickly^5–7^. Various reactors have been made that are capable to remove or use the minimum amount of compounds that involve the carbon oxides and hydrogen bonds to optimize the enthalpy or entropy for the chemical reaction under observation^8–10^. Large industrial applications are founded on the side. The propylene oxide is simply called epoxy propane and is included in the list of the organic compound. It is the chemical compound that is almost used in chemical reactions which are intermediate and widely used in the chemical industries to yield commercial products. It is also included in the list of 50 compounds that are widely used for the required production and annual demand is increased up to 14 billion pounds. The chemical reaction of propylene oxide with water produces propylene glycol which is largely used as an anti-caking agent, Antioxidant, Carrier, dough-strengthener, emulsifier, moisture-preserver, and a texturizer. The application of propylene glycol is found in making and caring the foods. The idea behind using the chemical reactions under the fluid dynamics problem might be whether someone is interested to increase the temperature or want to decline it according to their requirements. Moreover, to run the wind turbine machine, the enthalpy change needs special attention in recent works. While a certain compound reacts decomposes in water, releases energy, or absorbs energy (exothermic and endothermic reactions). To get a better advantage from the chemical reaction, it is always necessary that whatever the species are used in the reactors, they must react chemically with each other to give/take a certain amount of energy. According to collision theory, a sufficient amount of energy should be provided to a certain amount of reactants to perform the chemical reaction with each other^11–13^. Due to this reason, in the channel, sufficient activation energy should be provided to the reactants so that they can perform a precious chemical reaction under the component to give a certain amount of the products which can fulfill the researcher's requirement in the vessel or components^14–16^. It is also known that the thermal conductivity of a chemical mixture or base fluid can majorly affect the thermal distribution in the reactors^17–20^. The amount at which the chemical species are diffused in the reactor can majorly impact the rate of reaction. Optimizing the thermal and mass transfer parameters are the fundamental requirements of today's engineering industries^21–23^. The enhancement in thermal characteristics due to the chemical processes is the fundamental requirement in the engineering field and industrial sciences. Here, we are going to report the following literature review:

Thermal and mass transfer in a vertical-cavity heated below was examined numerically to form the correlation between the dynamical and thermal non-dimensional parameters^24^. Using the Soret and Dufour coefficients, a heated enclosure domain was numerically examined by double-diffusive impact with the mixing of Al_2_O_3_-H_2_O nanofluids^25^. An infinite vertical plate was observed for impacts of first-order homogeneous reaction for the unsteady flow for constant mass and heat distribution^23,24^. A study of magneto-hydro-dynamics MHD flow was done in account to observe a vertical stretchable plate with the permeable surface under the chemical reactions^26^. A novel study was performed by assuming that the plate contained in the uniform porous medium and continuously moved with constant speed in the presence of the magnetic field. Another investigation for consequences of chemical reactions as well as radiation on the unsteady MHD and free convection was done to observe the flow past through a semi-infinite moving plate in the presence of heat source or suction^27–31^. With the implementation of thermo-analytical techniques, the decomposition of barium carbonate was investigated and found the activation energy of barium carbonate was for the initial stage using the gravimetric data^32^. The flow of heat and the mass transfer in a packed bed reactor was investigated by^30^ under the conditions of reacting and non-reacting species. In the article, the most ordinary sample of the reactor was discussed which was containing cooled walls.^33,34^ Analyzed the consequences of homogeneous and heterogeneous reactions in the chemical engineering problems on the diffusivity of species in the nanofluids under the presence of the magnetic field. The fluid under consideration was an idealized fluid i.e. the fluid which is not made of electrons. The mass and thermal diffusion were examined, where the intention was to compare and contrast the mass diffusion and the thermal conduction for the two-dimensional plug flow concerning the convective property^35,36^. The counter flow diffusion of chemical species hydrogen and oxygen was studied by^37^. In the article, the most critical points related to thermodynamic and transport properties were discussed. A theoretical study was done on the consequences of the homogeneous and heterogeneous chemical mixture over the diffusion of species in the chemical mixture where the surface is moving with constant speed or velocity^38^. One dimensional steady-state model was developed on account of a tubular reactor in the procedure for naphtha cracking^39^. About 90 species with 543 reactants were used to implement a free-radical scheme. The aim behind the study was to maximize the operating. Often the field of computational fluid dynamics CFD shows the complex picture of the stable radial flows^40^. It describes the local heat transfer can be connected with the local field of flow. It was also deducted that some heat transfer parameters are less or not affected by the pressure as well as wall temperatures^41^. With the implementation of finite element-based software COMOSL Multiphysics 5.4, various achievements were gained and successfully describe the characteristics of fluid dynamics problems with the help of fluidic and thermal parameters in the rectangular channels with and without obstacles with screen boundary conditions. The results were displayed with graphs and tables, similar to those reported in Refs.^42–46^. The numerical results contrast with the asymptotic solution gained by the implementation of the screen boundary in problems.

In the current research article, we are going to focus on the heat and mass transfer in the Multicomponent tubular reactor under the decomposition of propylene oxide in water with the standard temperature of 25 °C with different activation energies, Reynolds number, and thermal conductivity of the mixture. Mainly, we are focusing on the full decomposition of the compound propylene oxide in water and suggest the critical Reynolds number for the problem to give a maximum decomposition of propylene oxide. Then we determine the total enthalpy change of the system against the tubular reactors by fixing two of the three parameters. The maximum total enthalpy change will be calculated and suggested the required parameters for it. Finally, the Sherwood-Nusselt number relationship will be focused on through the graphs.

## Methodology

### Physical description of tubular reactors and parameters selection

**Figure 1:**
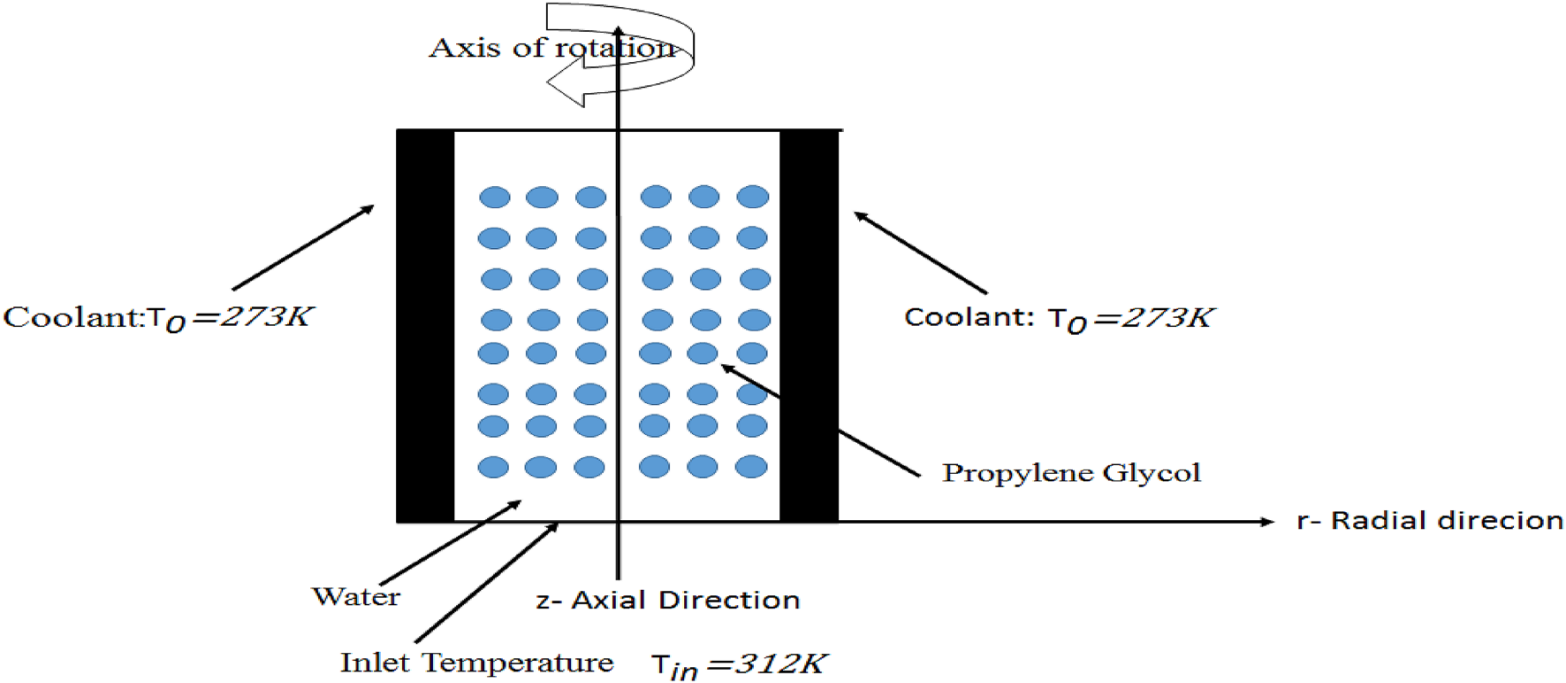
Schematic representation of multicomponent tubular reactor with isothermal cooling jacket and inlet temperature.

**Figure 2:**
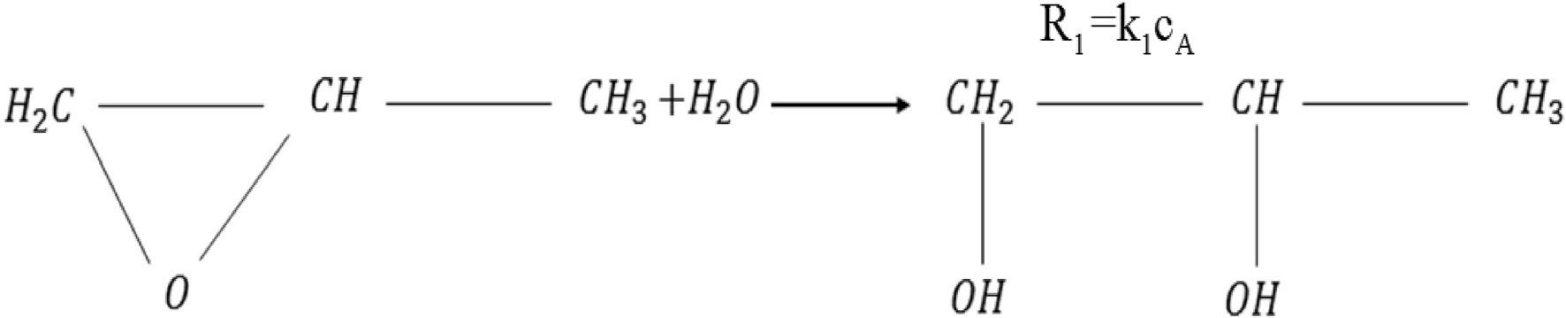
Chemical reaction of propylene oxide with water to yield propylene glycol.

A Multicomponent tubular reactor of length *L* = 1 m with radius *R*_*a*_ = 0.1 m is under observation for measuring parameters of mass and heat transfer see Fig. 1. The component is carried cooling jackets at the outer surface with a constant temperature of *T*_*0*_ = 273 K. The propylene oxide is disintegrated in water to give propylene glycol in which the water is being in excess and the reactor is keeping at the reference temperature of *T*_*ref*_ = 293 K. The chemical reaction is of the first order, irreversible, and exothermic reaction that releases the energy of $\Delta H = - 84,666\;{\text{J/mol}}$. Since the water is present in the excess so the rate of reaction r is depending on only on the concentration of propylene oxide. We are giving this hydrolysis reaction equation in Fig. 2 and Arrhenius Eq. (1) to measure the rate of reaction as follow:

Where R_1_ and is the rate of reaction in mol/s and *k*_*1*_ is rate constant given by the Arrhenius equation:

1 $$k_{1} = Ae^{{ - \frac{E}{RT}}}$$

where *R* is the universal gas constant.

Normally, to break the strong bonding of the propylene oxide requires threshold energy (Activation energy *E*) in the range of 75,000 to 76,000 in J/mol. For the current problem, on the trial basis, the activation energy is taken in the range from 75,000 to 80,000 J/mol with fixed Arrhenius constant *A* (frequency factor) of 16.96 × 10^12^. Moreover, the initial amount of propylene oxide *c*_*po*_ is tested from 2 to 3% to the amount of water to give a diluted mixture for transportation. The inlet of the tubular reactor is facing a constant temperature of *T*_*in*_ = 312 K. The consequences of the hydrolysis reaction over the propylene oxide under the laminar fluid flow with the constant viscosity, we are developing the Reynolds number in the range from 100 to 1000. That range will enhance the total flow rate at the inlet of the reactor in the range from 3.14 × 10^−6^ to 3.14 × 10^−5^ in m^3^/l. Equations (2) and (3) describe the computation of total flow rate and Reynolds number respectively.

2 $$u_{in} = \frac{{v_{in} }}{{\pi R_{a}^{2} }}$$

3 $${\text{Re}} = \frac{{\rho_{w} u_{in} L_{c} }}{{\mu_{w} }}$$

where L_c_ is the characteristic length of the tubular reactor. Generally, the Reynolds number can also be written in terms of the total flow rate.

4 $${\text{Re}} = \frac{{\rho_{w} v_{in} L_{c} }}{{\mu_{w} \pi R_{a}^{2} }}$$

For further information about the parameters used in the problem refer the Table 1.

**Table 1:** Global parameter and variational parameters of the problem.

Symbols	Value	Description
*E*	75,000–80,000 (J/mol)	Activation energy
*A*	16.96 ${{ \times }}$ 10^12^ (1/h)	Frequency factor
*U*_*k*_	1300 (W/m^2^/K)	Overall heat-transfer coefficient
*k*	0.559–0.799 (W/m/K)	Thermal conductivity mixture
*T*_*in*_	312 (K)	Inlet temperature
*T*_*0*_	273 (K)	Inlet temperature of the coolant
$\Delta H$	−84,666 (J/mol)	Enthalpy of reaction
*V*_*in*_	${3}{{.14 \times 10}}^{{ - 6}} \to {3}{{.14 \times 10}}^{{ - 5}}$	Total flow rate
*U*_*in*_	${\text{v}}_{{{\text{in}}}} {{/\pi R}}_{{\text{a}}}^{{2}}$	Average flow velocity
*P*_*R*_	2–3%	Percentage amount of propylene oxide in water
*c*_*po*_	P_R_ ${{ \times }}$ c_w_	Propylene oxide concentration, inlet
*c*_*w*_	43,210 mol/m^3^	Water concentration, inlet
*R*_*a*_	0.1 (m)	Reactor radius
*L*	1 (m)	Reactor length
*m*_*po*_	58.095 (g/mol)	Molar weight, propylene oxide
*m*_*w*_	18 (g/mol)	Molar weight, water
*m*_*pg*_	76.095 (g/mol)	Molar weight, propylene glycol
$\rho_{po}$	830 (kg/m^3^)	Density, propylene oxide
$\rho_{w}$	1000 (kg/m^3^)	Density, water
$\rho_{pg}$	1040 (kg/m^3^)	Density, propylene glycol
$\mu_{ref}$	0.001 (Pa ${{ \times }}$ s)	Reference dynamic viscosity, water
*T*_*ref*_	293 (K)	Reference temperature viscosity
*cp*_*m*_	75.36 (J/mol/K)	Molar heat capacity, water
*Re*	100–1000	Reynolds number

### Mesh independent test

Whenever we are trying to solve any type of CFD problem with the computational methods it needs a meshing process to discretize the domain of interest. In fact, we are finding a discontinuous solution space with the use of elements instead of continuous solution space. It is generally known that the degree of accuracy depends upon the number of elements used in the meshing process.

It is the procedure to achieve an approximated result of a selected parameter by refining the mesh from coarse mesh to extra fine mesh. No matter what structure of elements is used in the meshing process but it needs to reduce the convergence error in the results of two successive approximations of the chosen parameter. A point comes where the computational results of the chosen parameter stopped improving further than the previous one. This is the point, where the problem of interest has a high level of accuracy.

**Figure 3:**
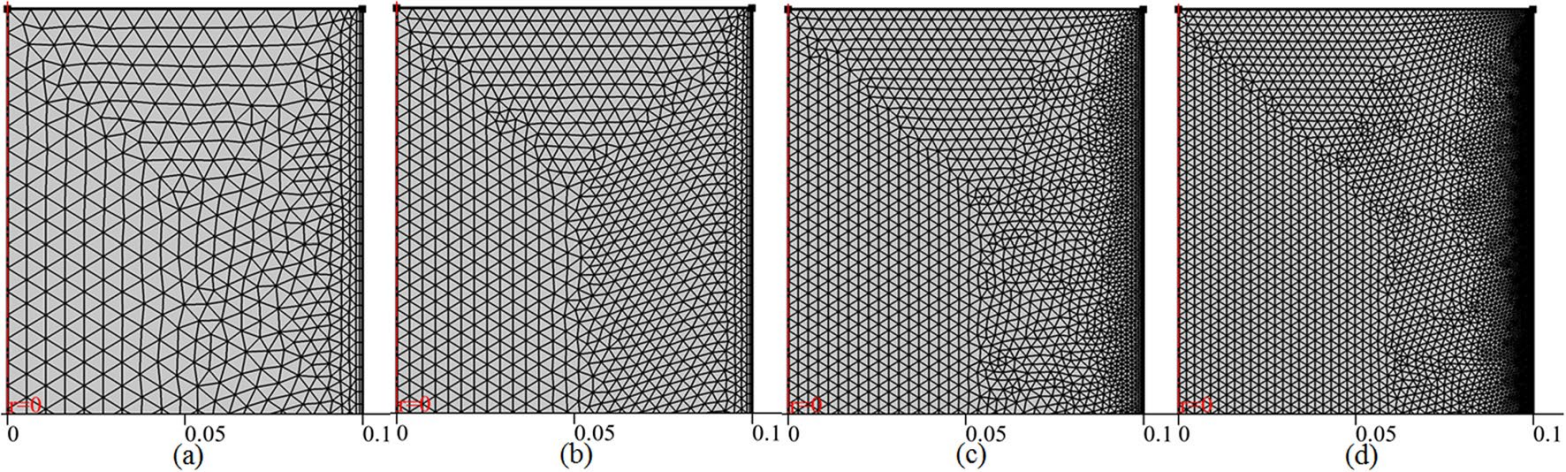
Different trials of the meshes at the outlet of axisymmetric channel for mesh-independent studies **(a)** normal mesh, **(b)** fine mesh, **(c)** finer mesh and **(d)** extra fine mesh.

**Figure 4:**
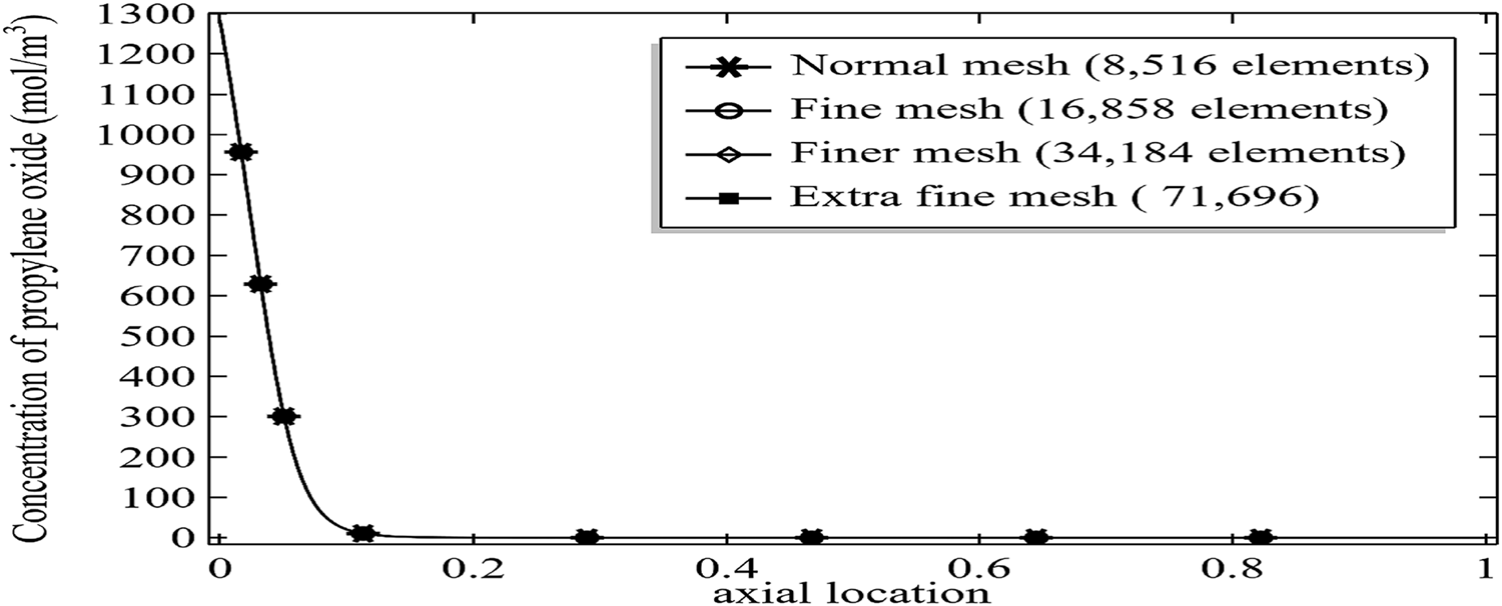
Decomposition of propylene oxide at different number of elements at Re = 100, E = 75,000, k = 0.599, and Initial concentration of 3%.

For the current problem of fluid, thermodynamics and chemical engineering we tried to find the solution by using irregular triangular meshes see Fig. 3. The mesh is four times refined from normal mesh to extra fine mesh and the computational result for the concentration of deactivation of propylene oxide is presented in Fig. 4. The graph shows even using the normal mesh of 8,516 elements generates a good result with sufficient accuracy when compared with the extra fine mesh having elements 71,696. For an unbiased solution and to keep higher and higher accuracy of the results, here we are going to compute the all results using an extra-fine mesh which will take about 3 h 20 min on my computer using the Comsol multiphysics 5.4.

### Governing equations and boundary conditions

Due to the chemical reaction of an exothermic reaction in the tubular reactor, it is essential to avoid explosion. Therefore, the tubular reactor is folded with the cooling jacket which is continuously supplied the cooling environment inside the reactor. Using the COMSOL Multiphysics 5.4 software the numerical solution is being simulated for the momentum balance, mass balance, and energy balance governing equations with the use of a chemical engineering interface to develop the chemical reaction for the three species.

To model mass and heat transfer in the tubular reactor, we are assuming that the three species are identical in terms of diffusivity. The modeling and simulation in the tubular reactor can be obtained by six governing partial differential equations, to model the laminar flow we have one mass and two momentum balance equations, and we have one balanced equation for each of the material balance for the species, energy balance for the core of the reactor and the cooling jacket. Since the channel under investigation possesses a rotational symmetry, therefore the numerical solution of all the governing partial differential can be modeled for a 2D axisymmetric axis.

The equations solved for the cooling jacket is an ordinary differential equation and is used as the boundary condition around the surface of the tubular reactor. All other differential equations are governed by partial differential equations: The equation and the boundary conditions are described below as well as all other dimensional and non-dimensional parameters.

5 $$\frac{1}{r}\frac{{\partial (rv_{r} )}}{\partial r} + \frac{{\partial (v_{z} )}}{\partial z} = 0$$

6 $$\rho \left\{ {\frac{1}{r}\frac{\partial }{\partial r}(rv_{r} v_{r} ) + \frac{\partial }{\partial z}(rv_{r} v_{z} )} \right\} = \mu \left\{ {\frac{2}{r}\frac{\partial }{\partial r}\left( {r\frac{{\partial v_{r} }}{\partial r}} \right) + \frac{\partial }{\partial z}\left( {r\frac{{\partial v_{r} }}{\partial z}} \right) - 2\frac{{v_{r} }}{{r^{2} }} + \frac{\partial }{\partial z}\left( {\frac{{\partial v_{z} }}{\partial r}} \right)} \right\} - \frac{\partial p}{{\partial r}}$$

7 $$\rho \left\{ {\frac{1}{r}\frac{\partial }{\partial r}(rv_{r} v_{z} ) + \frac{\partial }{\partial z}(rv_{z} v_{z} )} \right\} = \mu \left\{ {\frac{1}{r}\frac{\partial }{\partial r}\left( {r\frac{{\partial v_{z} }}{\partial r}} \right) + 2\frac{\partial }{\partial z}\left( {\frac{{\partial v_{z} }}{\partial z}} \right) + \frac{1}{r}\frac{\partial }{\partial r}\left( {r\frac{{\partial v_{r} }}{\partial z}} \right)} \right\} - \frac{\partial p}{{\partial z}}$$

8 $$\rho c_{p} \left\{ {v_{r} \frac{\partial T}{{\partial r}} + v_{z} \frac{\partial T}{{\partial z}}} \right\} = k\left\{ {\frac{\partial }{\partial z}\left( {\frac{\partial T}{{\partial z}}} \right) + \frac{1}{r}\frac{\partial }{\partial r}\left( {r\frac{\partial T}{{\partial r}}} \right)} \right\} + ( - \Delta H) + R_{i}$$

9 $$\rho c_{p} \left\{ {v_{r} \frac{\partial C}{{\partial r}} + v_{z} \frac{\partial C}{{\partial z}}} \right\} = k\left\{ {\frac{\partial }{\partial z}\left( {\frac{\partial C}{{\partial z}}} \right) + \frac{1}{r}\frac{\partial }{\partial r}\left( {r\frac{\partial C}{{\partial r}}} \right)} \right\} - R_{i}$$

10 $$- \frac{\partial T}{{\partial r}} = \frac{{U_{k} }}{k}(T - T_{c} )$$

where $\rho$, $k$ and $\mu$ are the density thermal conductivity and viscosity of the mixture and the Eq. (10) will be working as a boundary condition. Finally, we are specifying the boundary conditions.

At inlet *z* = 0,

$$c_{i} (r,0) = initial\,\, concentration = c_{i0} , T(r,0) = T_{0} , v_{z} = v_{in}$$

At outlet *z* = *L*

$$\frac{{\partial c_{A} }}{\partial r}(r,L) = 0,{ - }\frac{\partial T}{{\partial r}}(r,L) = 0,{\text{ p = 0}}$$

The total enthalpy of the system can be measured by:

11 $$H_{t} = H_{f} + K.E$$

12 $$H_{f} = H_{ref} + \Delta H$$

where $H_{f}$ and K.E are the enthalpy of the formation and kinetic energy of molecules respectively. Here $H_{ref}$ is the reference enthalpy and assumed zero. Local Nusselt number, Sherwood number, and Prandtl number are defined in their usual ways:

13 $$Nu_{z} = \frac{Convective\,\, heat\,\, transfer}{{Conductive\,\, heat\,\, transfer}} = \frac{{U_{k} z}}{k}$$

14 $$Sh = \frac{{{\text{Convective\,\, mass\,\, transfer\,\, rate}}}}{{\text{{Diffusion\,\, rate}}}} = \frac{h}{D/L}$$

and

15 $$\Pr = \frac{{{\text{Momentum\,\, diffusivity}}}}{{{\text{Thermal\,\, diffusivity}}}} = \frac{{\mu c_{p} }}{k}$$

where $h$ is the convective mass transfer coefficient (m/s), D is the mass diffusion (m^2^/s), L is the length of the reactor (m).

### Validation and comparison

Here, we are making efforts to compare the numerical results by the mass-heat transfer analogy in the correlation provided by the Churchill-Bernstein equation^47–50^. Originally, the Churchill-Bernstein Eq. (16) is the relation containing surface averaged Nusselt number, Reynolds number, and the Prandtl number.

16 $$Nu_{D} = 0.3 + \frac{{0.62Re_{D}^{1/2} Pr^{1/3} }}{{[1 + (0.4/Pr)^{2/3} ]^{1/4} }}[1 + \left( {\frac{{Re_{D} }}{282000}} \right)^{5/8} ]^{4/5}$$

The equation is valid where the Reynolds number and Prandtl number are frequently available and the product of these two might cover the criteria $RePr > 0.2$. With the use of the mass-heat transfer analogy, the Nusselt number and the Prandtl number would be replaced by the Sherwood number and the Schmidt number respectively. The correlation formed described by the Eq. (17)

17 $$Sh_{D} = 0.3 + \frac{{0.62Re_{D}^{1/2} Sc^{1/3} }}{{[1 + (0.4/Sc)^{2/3} ]^{1/4} }}[1 + \left( {\frac{{Re_{D} }}{282000}} \right)^{5/8} ]^{4/5}$$

**Figure 5:**
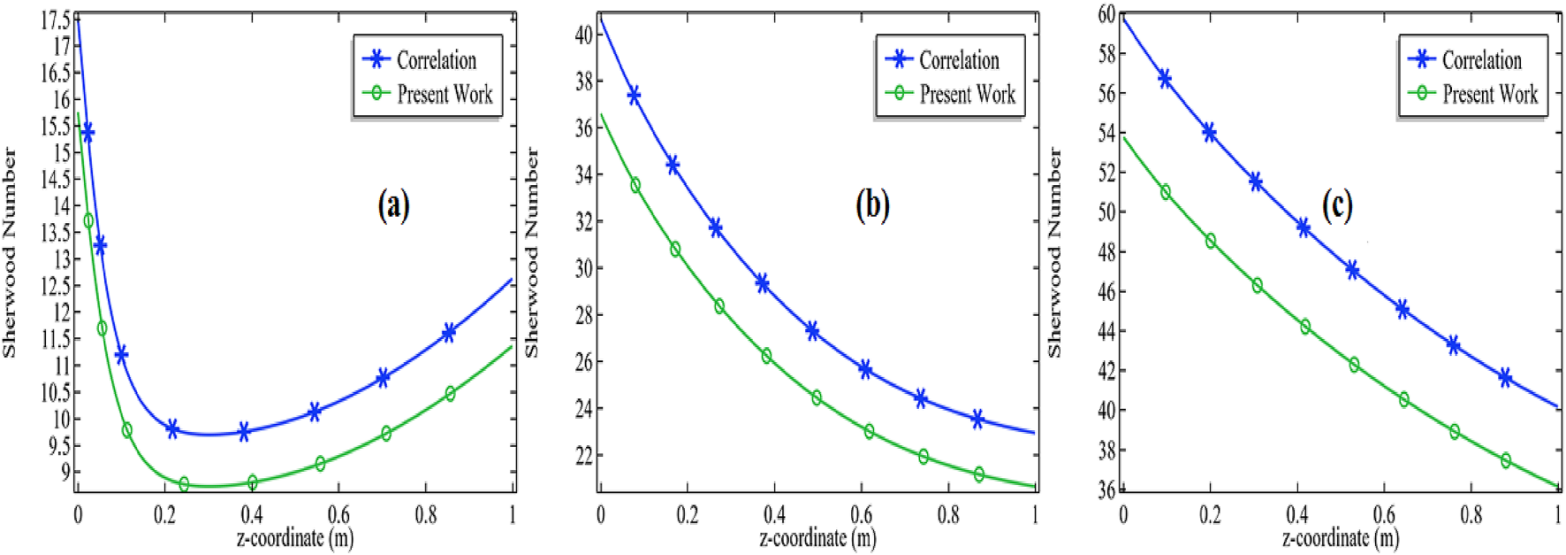
Comparison of Sherwood number with Churchill-Bernstein correlation at E = 76,000 with 2% initial concentration of propylene oxide for **(a)** *Re* = 100, **(b)** *Re* = 500 and **(c)** *Re* = 1000.

**Figure 6:**
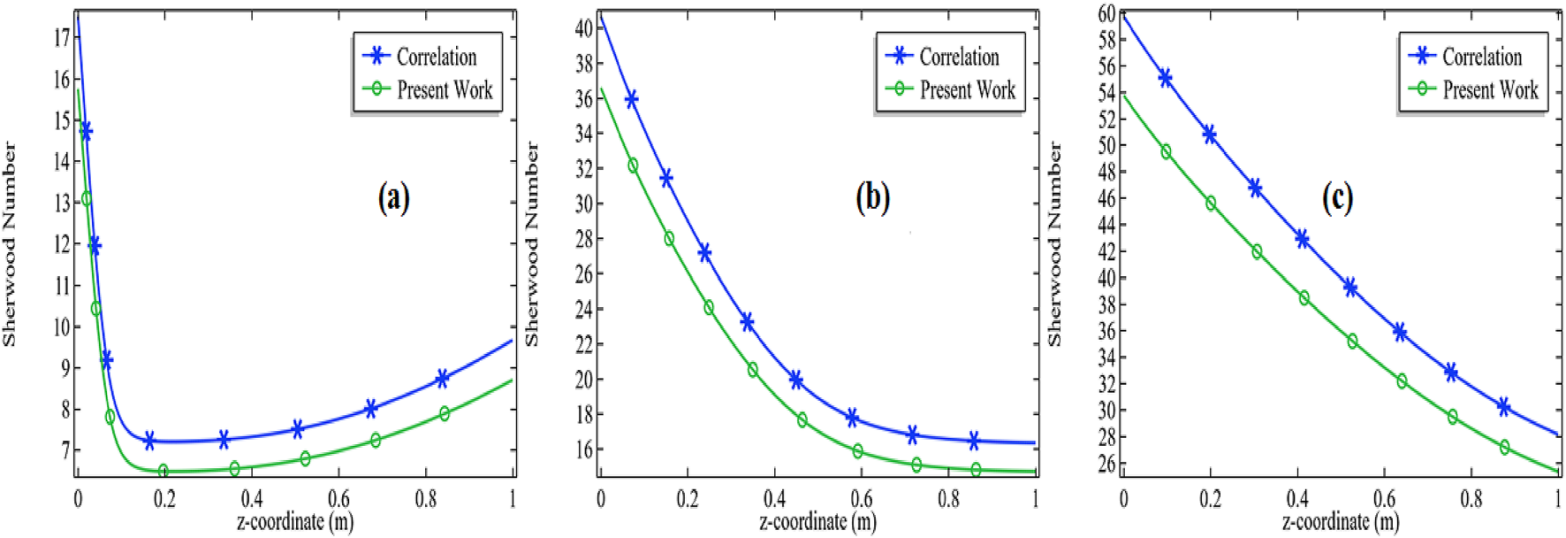
Comparison of Sherwood number with Churchill-Bernstein correlation at E = 76,000 with 3% initial concentration of propylene oxide for **(a)** *Re* = 100, **(b)** *Re* = 500 and **(c)** *Re* = 1000.

The correlation (Eq. 17) is tested to compute the left-hand side of the equation by the actual definition of the Sherwood number i.e. it is the ratio between the convective mass transfer rate to the diffusion rate and finally compare the results for *Re* = 100, 500, and 1000 with the activation energy *E* = 76,000 J/mol for the initial concentration of the propylene oxide with 2% and 3% see Figs. 5a–c and 6a–c.

These results show the stability of the computational results gained through the COMSOL Multiphysics 5.4. The results are also showing due to an increment in the initial concentration of the chemical species, the comparison between the computational results and the correlation is getting closer and closer. Also, the computational results got more fluctuation with the correlation due to the increase in initial velocity or Reynolds number of a chemical mixture. The distinction in the results is due to the reason that the current model is confirming with the analogy. The analogy is that a replacement of the Prandtl number can be made with the Schmidt number when dealing with the mass transfer problems. Also, it is explained in the literature that your results will obtain an accuracy of 80% when you compare your results with the correlations^43–45^. Surely, we can also presage from our simulation that accuracy can be improved when the chemical reactions are performing at the lower velocities so that the diffusion of the mixture remains under control and better accuracy will be achieved. But it is not a particular reason might be other factors are involved.

18 $$Sh_{D} = 2 + 0.552Re_{D}^{1/2} Sc^{1/3}$$

**Figure 7:**
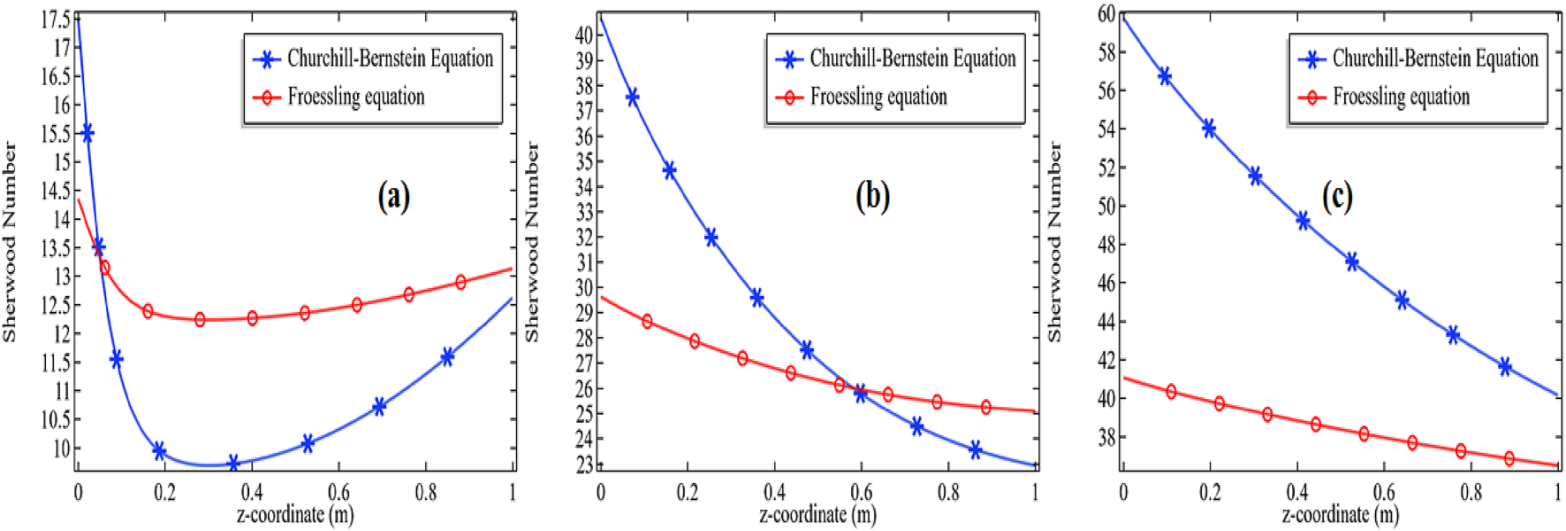
Comparison of Sherwood number from Churchill-Bernstein with Froeslling equation at E = 76,000 J/mol with 3% initial concentration of propylene oxide for **(a)** *Re* = 100, **(b)** *Re* = 500 and **(c)** *Re* = 1000.

Moreover, on comparing the two correlations of the Churchill-Bernstein equation and the Froessling^47^ Eq. (18) with our numerical results see Fig. 7a–c, we come to know these two correlations are also agreed with each other to some extent.

## Results and discussion

### Deactivation *of *propylene relevant to the parameter

**Figure 8:**
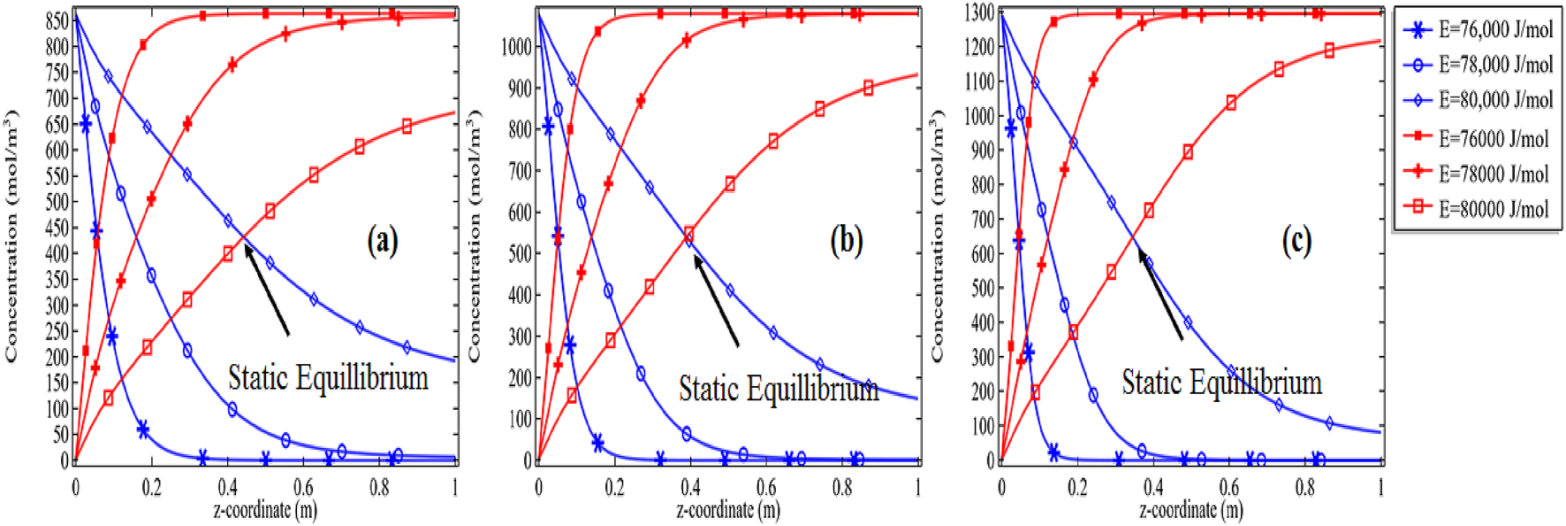
Concentration of propylene oxide v/s the concentration of propylene glycol for *Re* = 100 at different activation energies with initial concentration **(a)** 2%, **(b)** 2.5% and **(c)** 3%.

We tried a hydrolysis process on propylene oxide to break it in the water by providing sufficient activation energies of 76,000 J/mol, 78,000 J/mol, and 80,000 J/mol. In Fig. 8a–c the decomposition of propylene oxide and the formation of propylene glycol is described with the initial concentration of 2%, 2.5%, and 3% at *Re* = 100. For any particular case, we can see the rate of reaction is increasing or decreasing along the length of the tubular reactor at the fixed rate for deactivation and formation respectively. It is clear in the graphs of Fig. 8a–c for *Re* = 100 with *E* = 76,000 J/mol and 78,000 J/mol, full decomposition of propylene oxide and formation of propylene glycol are achieved. The location in the tubular reactor where these graphs are intersecting for particular activation energy E is the equilibrium points where the concentration of both molecules is equal. Also, the time to reach these locations is increasing with the increase in activation energy. With the increased initial concentration of propylene glycol, the deactivation of propylene oxide is decreased see cases of *E* = 80,000 J/mol in Fig. 8a–c, the percentage decrease in propylene oxide is about 53% at *Re* = 100 J/mol in Fig. 8a, in Fig. 8b the percentage decrease is about 60% and in Fig. 8c the percentage for deactivation is about 67%. In short, the percentage deactivation of the propylene oxide is a function of initial concentration i.e. more you add the compound in the solution greater will be the percentage of deactivation of the amount.

**Figure 9:**
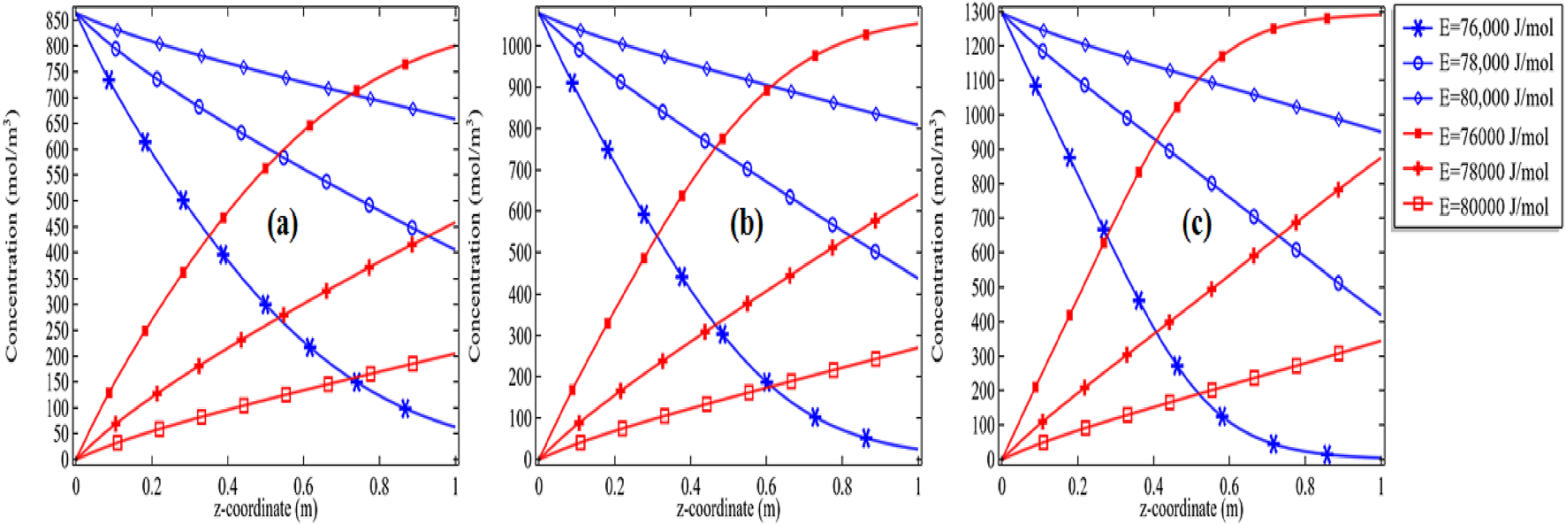
Concentration of propylene oxide v/s the concentration of propylene glycol for *Re* = 500 at different activation energies with initial concentration **(a)** 2%, **(b)** 2.5% and **(c)** 3%.

**Figure 10:**
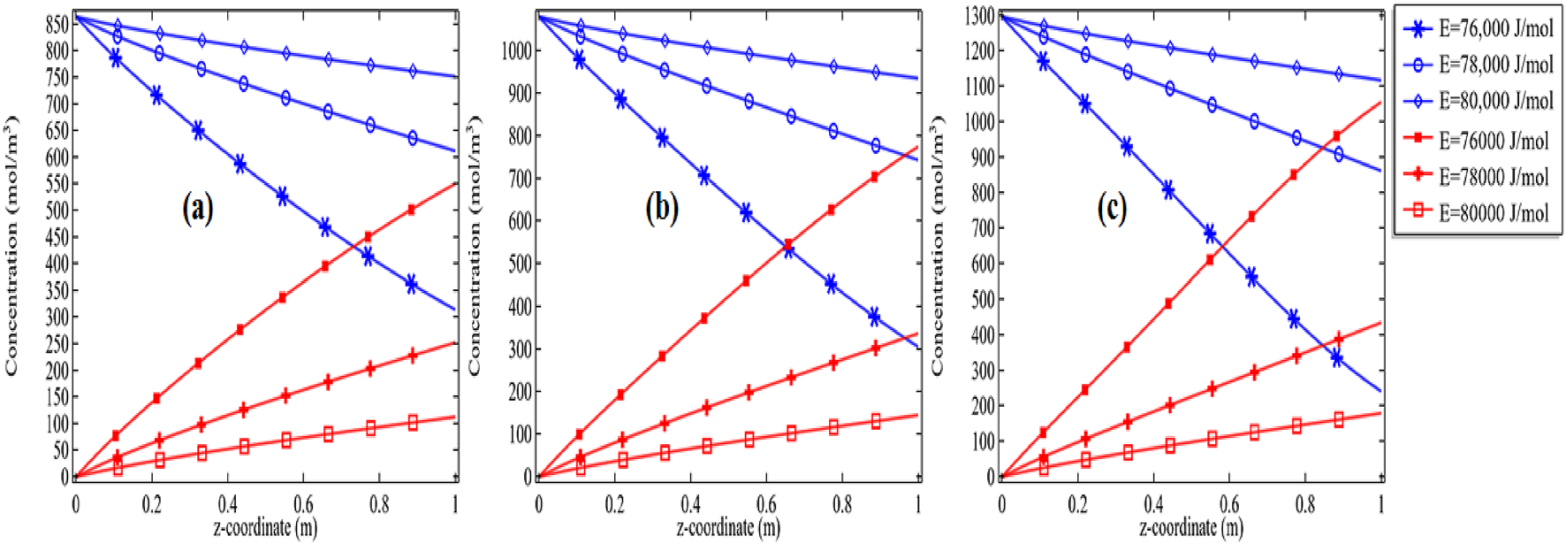
Concentration of propylene oxide v/s the concentration of propylene glycol for *Re* = 1000 at different activation energies with initial concentration **(a)** 2%, **(b)** 2.5% and **(c)** 3%.

We also produced the graphs for *Re* = 500 and *Re* = 1000 where we showed the pattern for the concentration for deactivation of propylene oxide and propylene glycol with the activation energy of 76,000 J/mol, 78,000 J/mol, and 80,000 J/mol along with the initial concentration of 2%, 2.5%, and 3% respectively see Figs. 9a–c and 10a–c. Now, all graphs 8(a–c)–10(a–c) are showing with the increase in Reynolds number the formation of propylene glycol/deactivation of propylene oxide is significantly decreasing with the increase in activation energy as well as with the initial concentration of propylene oxide. Often, it was thought that providing too much activation energy to let a chemical reaction happen, will produce a good quantity of the product. But our simulation has shown that with the increase in the activation energy more than normal activation energy, the goal to get the product earlier with good quantity cannot be achieved. For example, compare the cases of *Re* = 100, *E* = 76, 000 J/mol and initial concentration of 2% in Figs. 8a, 9a and 10a with each other. In case Fig. 8a propylene oxide is decomposed completely, in Fig. 9a propylene is decomposed about 94%, in the case of Fig. 10a the propylene oxide is deactivated about 64%. It means increasing the initial velocity of the solvent the chemical reaction among the molecules is disturbed. Moreover, with the increase in the initial concentration of the compound the amount to deactivate is increasing see the cases of Figs. 8a–c, 9a–c, and 10a–c for the fixed energy with the variation in initial concentration.

Finally, we are going to display the percentage deactivation in the amount of propylene oxide for all cases discussed for the current problem. The table is also including the variation of thermal conductivity of the mixture. Table 1 indicates that there was an insignificant impact of thermal conductivity of the mixture on the amount deactivation of the compound in the tubular reactor. From the Table 2, it is also evident that the maximum decomposition of the propylene oxide (99.8%) is achieved at *Re* = 100, *E* = 76,000 with *k* = 0.599 and minimum decomposition of the propylene oxide (13.1%) is achieved at Re = 1000, E = 80,000 with k = 0.799.

**Table 2:** Percentages of deactivation of propylene oxide.

*Pr*	*K*	*Re* = *100* *E* = 76,000 J/mol	*Re* = *100* *E* = 78,000 J/mol	*Re* = *100* *E* = 80,000 J/mol	*Re* = *500* *E* = 76,000 J/mol	*Re* = *500* *E* = 78,000 J/mol	*Re* = *500* *E* = 80,000 J/mol	*Re* = *1000* *E* = 76,000 J/mol	*Re* = *1000* *E* = *7*8,000 J/mol	*Re* = *1000* *E* = 80,000 J/mol
*2*	0.599	99.080	89.037	53.899	92.588	53.738	24.215	65.834	30.485	13.599
*2*	0.699	98.482	85.290	48.816	90.959	51.417	23.129	64.905	29.990	13.384
*2*	0.799	97.728	81.437	44.533	89.197	49.230	22.111	63.971	29.503	13.173
*2.5*	0.599	99.584	93.481	60.094	97.351	59.878	25.538	74.045	32.612	14.016
*2.5*	0.699	99.250	90.565	54.076	96.360	57.051	24.322	72.958	32.039	13.786
*2.5*	0.799	98.797	87.327	48.980	95.173	54.402	23.191	71.850	31.477	13.561
3	0.599	99.826	96.409	67.766	99.414	67.711	27.064	83.601	35.161	14.468
3	0.699	99.652	94.400	60.767	99.041	64.247	25.687	82.449	34.486	14.221
3	0.799	99.396	91.999	54.679	98.523	60.999	24.420	81.233	33.824	13.980

### Total enthalpy and maximum total enthalpy change in the system

By definition, the enthalpy of a system is the sum of the internal energy and the product of mass and volume. The product of pressure and volume causes the atmosphere for the system to do non-mechanical work. Generally, it is known that it is almost unlikely to determine the total enthalpy of the system. The reason behind this we do not know the total energy of the system and in short zero points of the system is undetermined. Assuming all factors generating the internal of the system are known as in our simulation then we could determine the total enthalpy and the change in enthalpy of the system. Applications of enthalpy are widely found in refrigeration and in combustion problems where the heat through vapors is found beneficial.

The heat and mass transfer are investigating in the current problem through the decomposition of propylene oxide and water (hydrolysis) to form propylene glycol. The reaction is an exothermic reaction and the standard rate of reaction is ${\Delta }H{\text{ = - 84,666 J/mol}}$**.**

**Figure 11:**
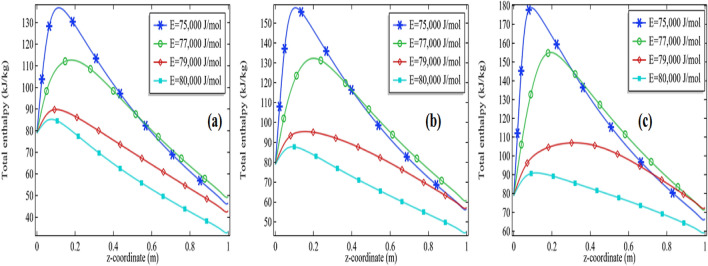
Measurement of total enthalpy in axial direction at different activation energies for *Re* = 100, *k* = 0.599 with **(a)** 2% initial concentration, **(b)** 2.5% initial concentration and **(c)** 3% initial concentration.

**Figure 12:**
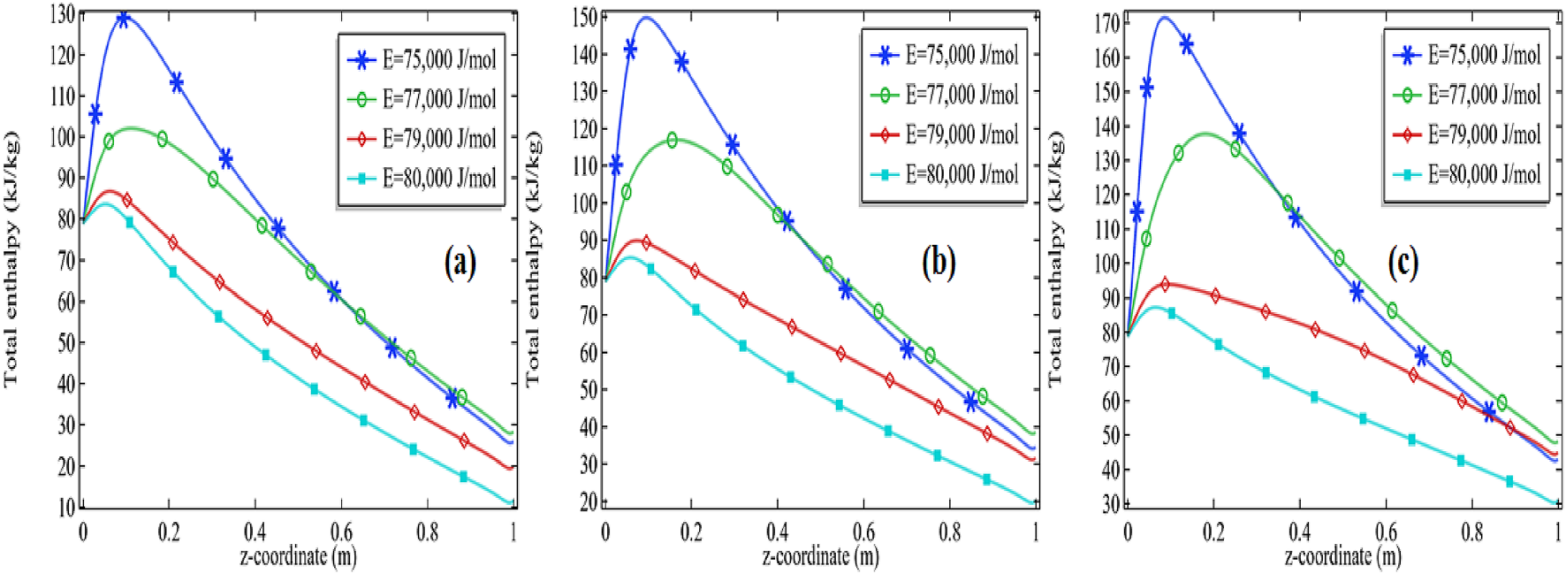
Measurement of total enthalpy in axial direction at different activation energies for Re = 100, k = 0.799 with **(a)** 2% initial concentration, **(b)** 2.5% initial concentration and **(c)** 3% initial concentration.

**Figure 13:**
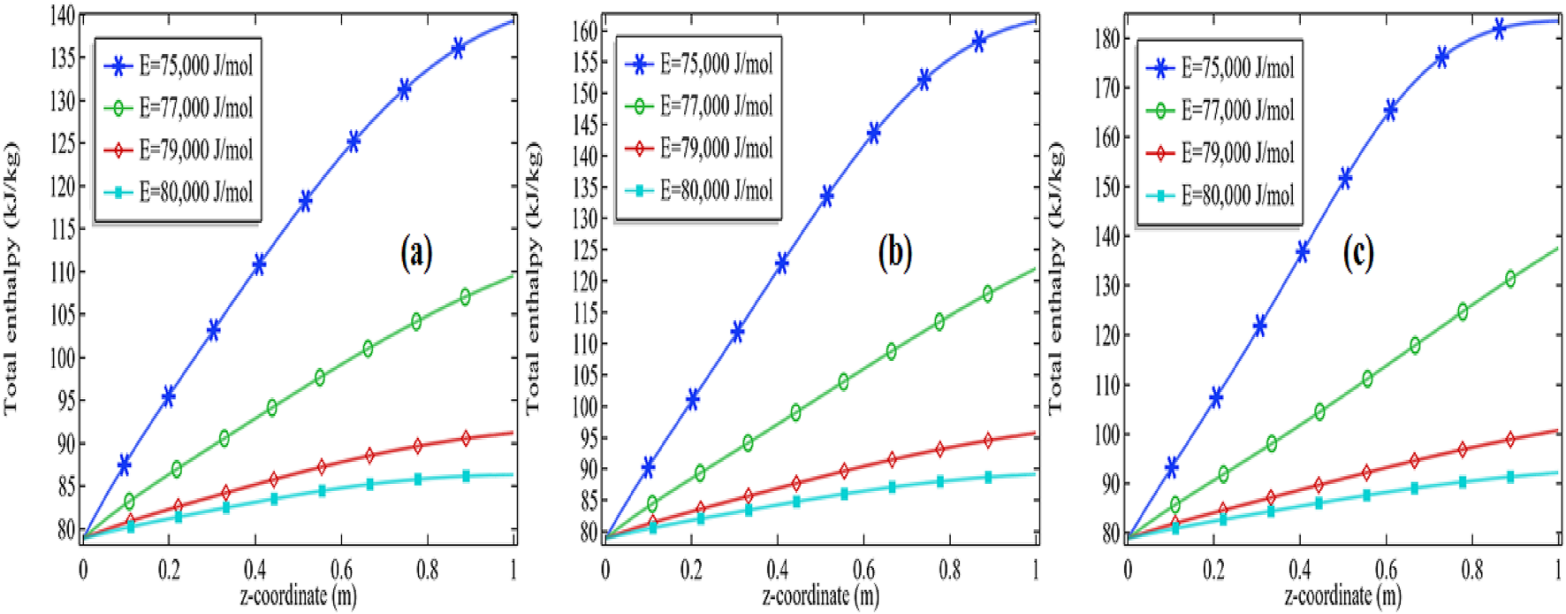
Measurement of total enthalpy in axial direction at different activation energies for *Re* = 1000, *k* = 0.599 with **(a)** 2% initial concentration, **(b)** 2.5% initial concentration and **(c)** 3% initial concentration.

**Figure 14:**
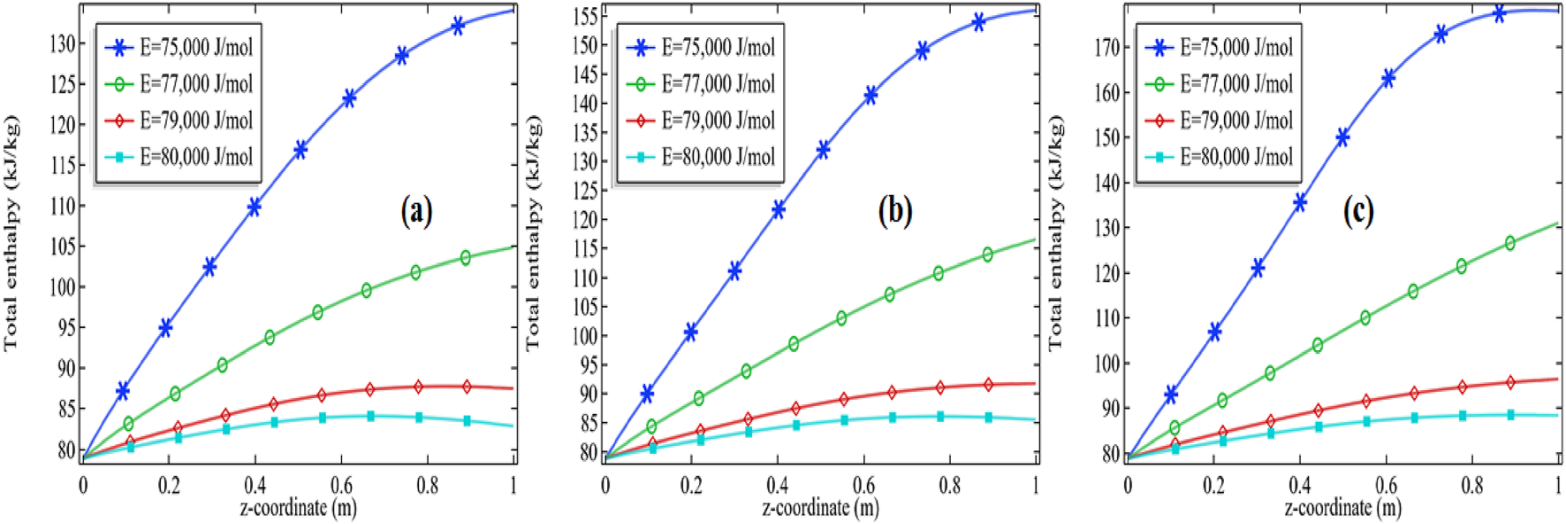
Measurement of total enthalpy in axial direction at different activation energies for *Re* = 1000, *k* = 0.799 with **(a)** 2% initial concentration, **(b)** 2.5% initial concentration and **(c)** 3% initial concentration.

The total enthalpy is presented in Figs. 11a–c, 12, 13 and 14a–c. The consequences of total enthalpy are presented by fixing the Reynolds number and thermal conductivity of the mixture with the variation in the activation energy and the initial concentration of the propylene oxide. It is evident from the graph by fixing the Reynolds number as well as the thermal conductivity of the mixture the total enthalpy is decreasing with the increase in activation energy *E*. For *Re* = 100 and k = 0.599, 0.799, the total enthalpy is increasing first attempts a maximum value then it is declined to see Figs. 11a–c and 12a–c. The total enthalpy is showing a positive response concerning the initial concentration i.e. more you add the compound greater the maximum enthalpy is generated. For example, if we increase the initial concentration from 2 to 3% it increases the total enthalpy about 33.3% for *Re* = 100 at *k* = 0.599 and 30.7% for *Re* = 500 at *k* = 0.799 see Figs. 11a–c and 12a–c. Although, by fixing *Re* = 100, the total enthalpy is increasing comparatively through the length of the tubular reactor see Figs. 11a and 12a.

Further, we are observing the graphs of total enthalpy with the increase of Reynolds number up to 1000. With the increase in Reynolds number, the total enthalpy of the system is increasing for a constant thermal conductivity of the mixture see Figs. 11a–c and 13a–c. For example, for 2% initial concentration with constant thermal conductivity *k* = 0.599 the maximum total enthalpy is increasing about 4% as we increase *Re* from 100 to 1000. Similar increments can be seen for 2.5% and 3%. Further, we are presenting the tables for the maximum enthalpy change in the system at different activation energies, initial concentration, and thermal conductivity of the mixture see Tables 3 and 4 for *Re* = 100 and *Re* = 1000 respectively. It is clear from these tables the maximum enthalpy change is decreasing with the increase in activation energy by fixing the thermal conductivity as well initial concentration. With fixed activation energy as well the initial concentration the maximum enthalpy change is showing a little bit negative response with the thermal conductivity of the mixture. Observing the particular cases in the tables, it is clear that the total maximum enthalpy change, for the case of *Re* = 100 is always greater in the case of Re = 1000. It is evident from the table that the total maximum enthalpy change is achieved at *Re* = 100, *E* = 75,000 J/mol, *k* = 0.559 with 3% initial concentration. We see the maximum total enthalpy near the inlet of the channel when Re = 100 in Fig. 11a–c but not in Fig. 13a–c. The reason behind this is obvious the increment in the initial flow velocity of the mixture but could be another reason for cooling temperature which is imposed on the rounded boundaries of the channel. Effect of those can be worked more effectively when the flow velocity is too much slow. It is one of the many reasons that the maximum total enthalpy for Re = 1000 is maximum near the outlet and minimum near the inlet of the reactor.

**Table 3:** The total maximum enthalpy change in KJ/mol for *Re* = 100 at different activation energies.

Pr	*k* (W/(m K))	*E* = 75,000 J/mol	*E* = 76,000 J/mol	*E* = 77,000 J/mol	*E* = 78,000 J/mol	*E* = 79,000 J/mol	*E* = 80,000 J/mol
2	0.599	235.05	234.52	232.26	226.49	215.31	200.04
2	0.699	234.52	233.49	230.06	222.26	209.07	193.93
2	0.799	233.89	232.25	227.54	217.88	203.51	189.26
2.5	0.599	253.22	253.20	251.96	247.76	237.35	218.42
2.5	0.699	252.78	252.45	250.26	243.92	230.05	209.19
2.5	0.799	252.27	251.50	248.16	239.51	222.78	201.83
3	0.599	271.30	271.62	271.13	268.59	260.69	241.69
3	0.699	270.89	271.06	269.92	265.62	253.76	229.64
3	0.799	270.44	270.35	268.36	261.91	245.98	218.96

**Table 4:** The total maximum enthalpy change in KJ/mol for Re = 1000 at different activation energies.

Pr	*k* (W/(m K))	*E* = 75,000 J/mol	*E* = 76,000 J/mol	*E* = 77,000 J/mol	*E* = 78,000 J/mol	*E* = 79,000 J/mol	*E* = 80,000 J/mol
2.0000	0.59900	225.60	210.08	195.48	184.91	177.80	173.09
2.0000	0.69900	225.50	209.98	195.41	184.86	177.77	173.07
2.0000	0.79900	225.41	209.89	195.35	184.82	177.74	173.06
2.5000	0.59900	248.24	229.09	207.46	192.05	182.15	175.83
2.5000	0.69900	248.13	228.93	207.35	191.98	182.11	175.80
2.5000	0.79900	248.02	228.80	207.25	191.92	182.07	175.78
3.0000	0.59900	270.83	252.81	222.36	200.33	186.94	178.73
3.0000	0.69900	270.74	252.60	222.20	200.23	186.88	178.70
3.0000	0.79900	270.65	252.42	222.05	200.15	186.83	178.67

### Relationship between Sherwood number and local Nusselt number

Sherwood number is well known as the ratio from convective mass transfer rate to diffusion rate. It is well known as the mass transfer Nusselt number. A change in the Sherwood number might be the reason for changes in convective mass transfer rate or diffusion rate across the boundary of a system. For heat and mass transfer to know the convective mass transfer rate and diffusion rate are essential. That's why the non-dimensional Sherwood number is used for the purpose. Similarly, the Nusselt number is known as the ratio between convective to conductive heat transfer. An increase in the Nusselt number means either the convection is enhanced in the domain or the conduction procedure is declined in the system. In the current section, we are going to focus on the relationship between the Sherwood number and the local Nusselt number along the length of the tubular reactor. For this purpose, we are presenting the graphs by fixing the Reynolds number (*Re* = 100 and *Re* = 1000) and thermal conductivity of the mixture (*k* = 0.599 and 0.799). The variation of the Sherwood number against the Nusselt number is checked by increasing the activation energy as well as the initial concentration of the compound propylene oxide.

**Figure 15:**
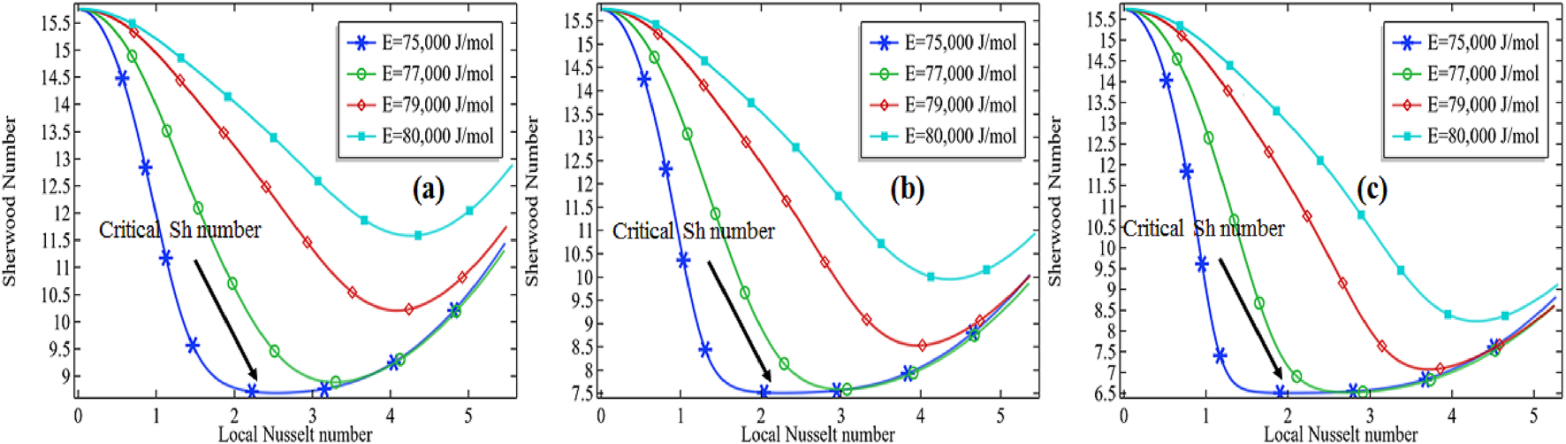
Sherwood number vs. local Nusselt number at different activation energies for *Re* = 100, *k* = 0.599 with **(a)** 2% initial concentration, **(b)** 2.5% initial concentration and **(c)** 3% initial concentration.

**Figure 16:**
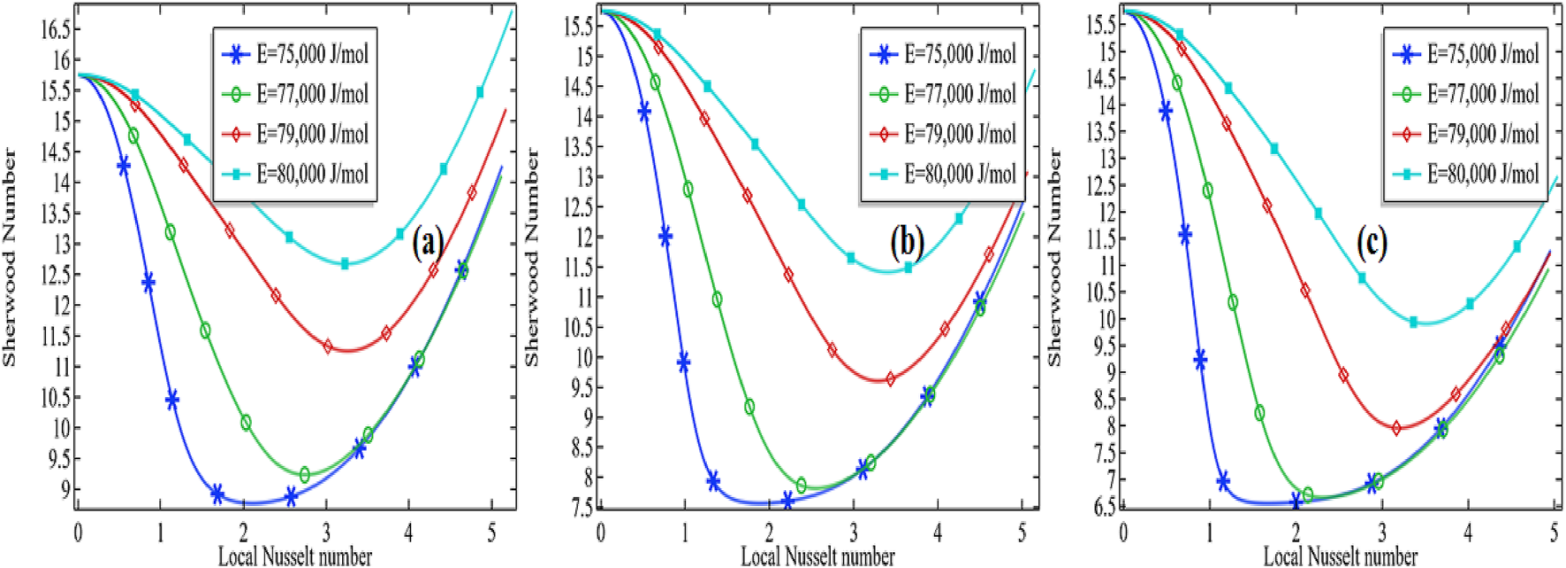
Sherwood number vs local Nusselt number at different activation energies for *Re* = 100, *k* = 0.799 with **(a)** 2% initial concentration, **(b)** 2.5% initial concentration and **(c)** 3% initial concentration.

**Figure 17:**
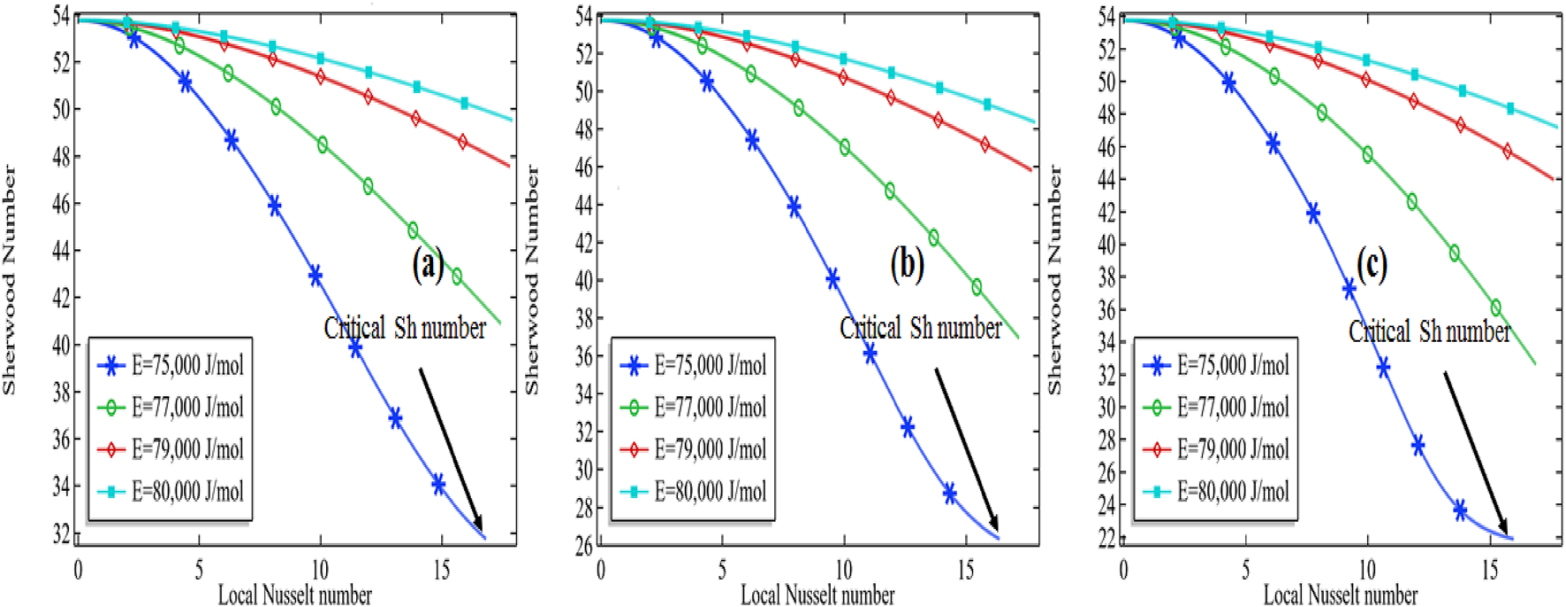
Sherwood number vs local Nusselt number at different activation energies for *Re* = 1000, *k* = 0.599 with **(a)** 2% initial concentration, **(b)** 2.5% initial concentration and **(c)** 3% initial concentration.

**Figure 18:**
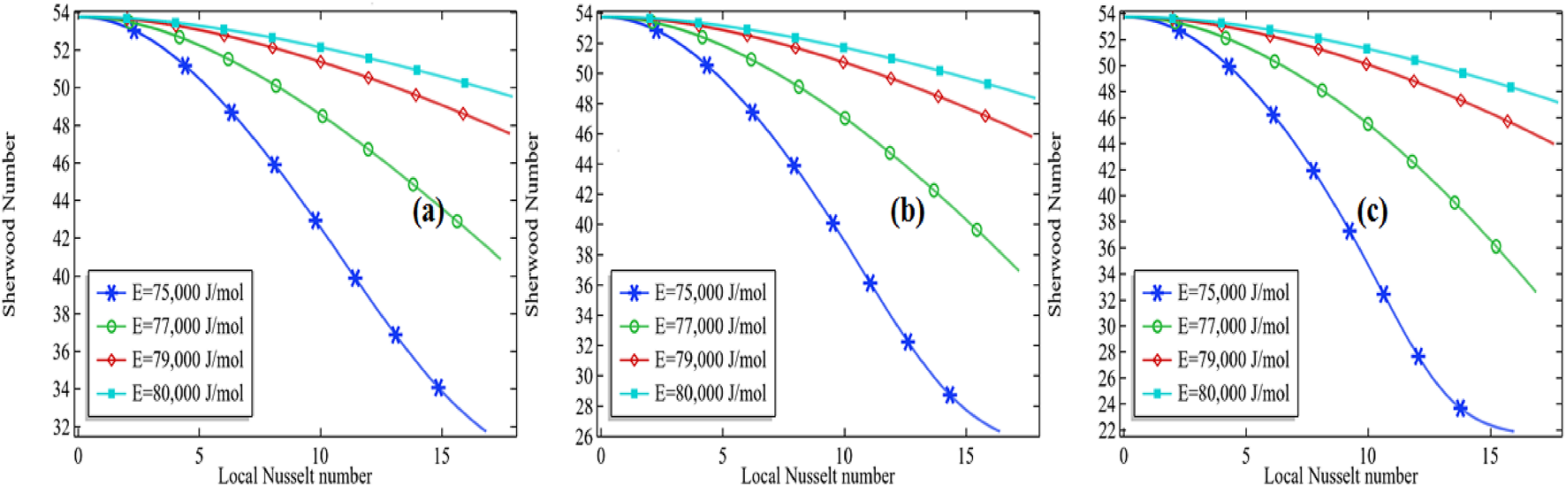
Sherwood number vs local Nusselt number at different activation energies for *Re* = 1000, *k* = 0.799 with **(a)** 2% initial concentration, **(b)** 2.5% initial concentration and **(c)** 3% initial concentration.

It is clear from the Figs. 15a–c, 16, 17 and 18a–c that the Sherwood number possesses a negative relationship with the local Nusselt number to some extent. For higher values of the Reynolds number, the relationship between the Sherwood number and the local Nusselt number is always negative whereas in the moderate Reynolds number the relationship cannot be judged. In Fig. 15a the Sherwood number is decreasing against the local Nusselt number for all activation energies then attempting a critical minimum value and then increased. The minimum value is declined with the decrease in activation energy. It means the diffusion rate is increasing with the increased activation energy or the mass transfer rate is decreasing with an increase in activation energy. Also, observing the graphs Fig. 15a–c, the critical minimum value is decreasing with the increase in the initial concentration of the propylene oxide. For example, an increase in the initial concentration from 2 to 3% for *Re* = 100 with *k* = 0.559, the critical minimum value is declined about 31.5% see critical minimum value in Fig. 15a,c. Similarly, for the case of *k* = 0.799, the critical minimum value is declined by about 33.3% see critical minimum value in Fig. 16a–c. We concluded Sherwood number is a little bit affected by the thermal conductivity of the mixture.

For high Reynolds number *Re* = 1000 in Figs. 17a–c and 18a–c, the Sherwood number is always decreasing with the increase in local Nusselt number for all activation energies. Keeping initial concentration as well as the constant Reynolds number, the critical minimum value of the Sherwood number is increasing with the increase in activation energy against the local Nusselt number. Moreover, the critical minimum value is decreasing more with the increase of initial concentration see the case of *Re* = 1000 with the activation energy of 75,000 J/mol with the initial concentration of 2% and 2.5% in Fig. 18a,b. We could conclude that the diffusion rate is very high and decreasing constantly or the mass transfer rate is very low in the case of a high Reynolds number. Meanwhile, the situation is altered with the domain in the case of moderate Reynolds number. The situation is depending upon the formation of propylene glycol. If it is formed early in the domain, the diffusion rate is decreasing quickly and then increases. But if the propylene is not fully formed then the diffusion rate is decreasing in the domain against the local Nusselt number. The minimum critical values are expressed in Table 5 for *Re* = 100 and Table 6 for *Re* = 1000, it is clear from the table the minimum critical value of the Sherwood number shows a little bit positive response against the thermal conductivity of the mixture for all the cases.

**Table 5:** Critical minimum value of Sherwood number against local Nusselt number for *Re* = 100.

P_R	*k*	*E* = 75,000	*E* = 76,000	*E* = 77,000	*E* = 78,000	*E* = 79,000	*E* = 80,000
2	0.599	32.187	36.496	41.349	45.372	48.330	50.406
2	0.699	32.832	37.240	42.109	46.115	49.054	51.004
2	0.799	33.606	38.119	43.002	46.988	49.809	51.415
2.5	0.599	26.763	31.028	37.209	42.575	46.484	49.186
2.5	0.699	27.324	31.774	38.006	43.348	47.227	49.906
2.5	0.799	28.012	32.662	38.940	44.253	48.096	50.535
3	0.599	22.371	25.478	32.658	39.547	44.533	47.920
3	0.699	22.820	26.181	33.492	40.357	45.299	48.654
3	0.799	23.355	27.033	34.475	41.302	46.194	49.492

**Table 6:** Critical minimum value of Sherwood number against local Nusselt number for *Re* = 1000.

*Pr*	*k*	*E* = 75,000	*E* = 76,000	*E* = 77,000	*E* = 78,000	*E* = 79,000	*E* = 80,000
2	0.599	9.7042	10.570	11.846	13.326	14.361	14.922
2	0.699	10.027	11.061	12.463	13.788	14.585	15.044
2	0.799	10.347	11.519	12.948	14.070	14.737	15.135
2.5	0.599	8.1897	8.8876	10.073	11.844	13.691	14.607
2.5	0.699	8.4535	9.3409	10.769	12.689	14.109	14.789
2.5	0.799	8.7295	9.7984	11.429	13.287	14.348	14.915
3	0.599	6.9214	7.4238	8.3699	9.9949	12.421	14.217
3	0.699	7.1152	7.7862	8.9941	10.958	13.416	14.494
3	0.799	7.3267	8.1705	9.6311	11.870	13.868	14.668

## Conclusion

The heat and mass transfer in the reactor of unit length were investigated with the thermal decomposition of propylene oxide in water. The chemical reaction engineering module of COMSOL Multiphysics 5.4 was used to observe a chemical reaction in the Multicomponent tubular reactor containing a cooling jacket of the fixed temperature around the surface. To perform the first-order irreversible chemical reaction between the molecules of water and propylene oxide, activation energy in the range from 75,000 to 80,000 J/mol was tested with the standard enthalpy of reaction of $\Delta H = {\text{ - 84,666 J/mol}}$ and frequency factor *A* = 16.96 × 10^12^. The mass balance, momentum balance, and energy equations were coupled to establish the finite element simulation in COMSOL Multiphysics 5.4. The various simulations were obtained by altering the Reynolds number from 100 to 1000, the thermal conductivity of the mixture from 0.559 to 0.799; the initial concentration of the propylene oxide from 2 to 3% with, and activation energy from 75,000 to 80,000 J/mol. Water was being in excess in the mixture, therefore the total rate of reaction is depending upon the concentration of propylene oxide. The rate constant was given by the Arrhenius equation. The results were displayed through the graphs and tables for the deactivation of propylene oxide, formation of propylene glycol, total enthalpy, maximum total enthalpy change, and the Sherwood–Nusselt number relationship. We made the following conclusion points:

- Fixing other parameters in the current problem the decomposition of the propylene oxide decreases with the increase in the activation energy.
- The amount of deactivation of the propylene oxide possesses a negative relationship with the increase of Reynolds number
- With the increase in thermal conductivity of the chemical mixture, the deactivation of propylene oxide is a little bit affected.
- The maximum decomposition of the propylene oxide is achieved by about 99.8% at *Re* = 100, *E* = 76,000 J/mol with the *k* = 0.559. Whereas the minimum decomposition of about 13.1% is achieved at *Re* = 1000, *E* = 80,000 J/mol, and with k = 0.799
- The formation of the propylene glycol does majorly impacts the total maximum enthalpy change at the lower Reynolds number. The maximum total enthalpy is decreasing with the increase in activation energy keeping other parameters constant.
- For the lower Reynolds number, the total enthalpy first increases up to the maximum value then decreases throughout the length of the reactor. But for the high Reynolds number, the total enthalpy is always increased. Therefore, we suggest the application of decomposition of propylene oxide can be used in a turbine to create electricity.
- Sherwood number is always decreasing with the increase in local Nusselt number for the higher values of the Reynolds number whereas for low or moderate Reynolds number the relationship cannot be judged because the Sherwood number is decreasing first up to critical minimum value then increases. This means the diffusion rate is dominated over the mass transfer rate with the increase in Reynolds number.
- Sherwood's number is decreasing with the increase in activation energy. This means the convective mass transfer rate is dominating over the diffusion rate of propylene oxide.
- Sherwood number also shows a positive relationship with the increase in Reynolds number. It means the convective mass transfer rate is increasing with an increase in Reynolds number.
